# Retention and recruitment of general dentists in an adjunct teaching model—A pilot study

**DOI:** 10.1371/journal.pone.0181602

**Published:** 2017-07-17

**Authors:** Brian J. Howe, Verasathpurush Allareddy, Christopher A. Barwacz, I. Reed Parker, Cheryl L. Straub-Morarend, David C. Holmes

**Affiliations:** 1 Department of Family Dentistry, College of Dentistry and Dental Clinics, University of Iowa, Iowa City, Iowa, United States of America; 2 The Iowa Institute for Oral Health Research, College of Dentistry and Dental Clinics, University of Iowa, Iowa City, Iowa, United States of America; 3 Department of Orthodontics, College of Dentistry and Dental Clinics, University of Iowa, Iowa City, Iowa, United States of America; 4 Craniofacial Clinical Research Program, College of Dentistry and Dental Clinics, University of Iowa, Iowa City, Iowa, United States of America; Waseda University, JAPAN

## Abstract

**Purpose/Objectives:**

Retention and recruitment of part time clinical adjunct faculty members in dental education is becoming increasingly difficult as dental schools come to rely on this workforce for their increased involvement in clinical education. Contributing factors include full time faculty shortage, aging workforce, practice and student debt, practice and family commitments, and financial compensation. This study attempts to ascertain barriers to teaching so appropriate strategies can be formulated to address this issue.

**Methods:**

In the spring of 2016 an email survey was sent to current and former adjunct faculty members to ascertain demographics and retention and recruitment strategies. Descriptive analyses were completed for all variables in the sample.

**Results:**

Twenty nine of forty six subjects responded to the survey with a response rate of 63%. Subjects over the age of sixty comprised 55% with only 17% being under the age of forty five. Overall family and practice commitments along with compensation were the primary barriers to teaching part time. For new dentists, student loan debt was the primary barrier to teaching. Travel to teach was also a barrier as 70% of respondents drove 200 miles or less to the dental school.

**Conclusion:**

The study demonstrated that the aging part time work force is a great concern and new part time clinical adjunct faculty members must be recruited. Barriers to recruitment and retention of faculty must be considered and addressed to sustain this teaching model.

## Introduction

Dental Schools in the United States continue to face a shortage of full and part time faculty which place increased responsibility and stress on current faculty members to satisfy the need left by unfilled faculty positions. According to the American Dental Education Association (ADEA), there has been a decrease in the number of vacant budgeted faculty positions from 2008–09 to 2010–11 of 17% (275 and 227 respectively) [1]. This decrease primarily consists of full time faculty positions as there was a slight increase (3%) in part time vacant budgeted faculty positions. However, these numbers do not take into account the number of full or part time faculty positions that have been lost due to budget constraints. Clinical science faculty constitute the largest group affected by the loss of full or part time faculty positions due to budget constraints which rose from 59% in 2008–09 to 79% in 2010–11. Yet when only looking at part time faculty positions lost due to budget constraints, there has been an increase of 67% [1]. Little change has been seen in the average number of faculty openings per dental school from 2008–09 to 2010–11, which remains at 4 openings per school, and schools with 10 or more faculty openings has increased from 10 to 15 in the same time period [1]. As a dental educational system we are utilizing part time/adjunct clinical faculty more often to help ease the burden of educating students at a time when there is still a shortage of full time faculty [2]. Dental schools are relying more heavily on part time clinical faculty to teach dental students, as reported by Holmes et al., who found in a 2002 survey that 46% of faculty are part time in dental schools in the U.S. and Canada [3]. This problem is not unique to the United States or to dentistry as it is also seen in medical, nursing, and pharmacy schools in Canada and the United Kingdom [4–8]. Most part time clinical adjunct faculty also see patients in a private practice setting in addition to their teaching responsibilities. This puts an increased amount of stress on practitioners and the school as their primary priority is typically with their private practice.

This area of research is poorly reported and there is little published research on this topic. This study attempt to fill the gaps in this research topic. The aims of this study are multifocal: to ascertain 1) demographic data pertaining to current part time clinical faculty, 2) barriers to teaching, and 3) tools for retention and recruitment. It is imperative to have a firm grasp of the demographics as well as the barriers to part time clinical teaching to better prepare dental schools to develop and implement unique strategies for retention and recruitment and maintain a part time clinical teaching model.

## Methods

A total of forty six (39 male, 7 female) part time clinical adjunct faculty members at the University Of Iowa College Of Dentistry, Department of Family Dentistry were asked through email to fill out an online survey through an anonymous survey link. Internal Review Board (IRB) exemption was attained by the appropriate IRB process and committee.

Subjects were recruited for the pilot study from the Department of Family Dentistry, which has the largest number of part time clinical adjunct faculty members at the University Of Iowa College Of Dentistry. The current adjunct teaching model in the department requires clinical teaching one half to one full day of teaching per week for one to four quarters during the academic year. If this commitment cannot be made, practioners may choose to teach on an occasional basis when the department’s and practioners’ schedules allow. Inclusion criteria include: current part time clinical adjunct faculty or former (retired or no longer wish to teach) part time clinical adjunct faculty from the last five years. Part time was defined as clinical teaching one half to one day per week during the academic year. Incomplete surveys were excluded from the study. Subjects’ email addresses were requested and attained with permission from the Department of Family Dentistry. An initial email was sent out with the survey link along with two additional survey reminders sent in two week intervals. The survey was prepared using Qualtrics Survey Software (Version 08.2015, Qualtrics, Provo, UT, USA). The questions are displayed in Table 1.

**Table 1:** The survey questions.

**Survey Question**
What is your gender?
What is your age?
To which racial or ethnic group to you most identify with? White-Caucasian/African American/Hispanic/Asian/Native American/Pacific Islander/Other
Please select from the following that best describes your practice type: General Dentistry/ Endodontics/Oral and Maxillofacial Surgery/Oral and Maxillofacial Pathology/Oral and Maxillofacial Radiology/Orthodontics/Pediatric Dentistry/Periodontics/Prosthodontics
Please select from the Practice arrangement that most closely approximates your current practice arrangement: Solo private practitioner/Partnership/Associate-non owner/Group Interdisciplinary/Corporate Practice/Public Health Clinic/Academic/Emeritus Faculty/Retired
If retired, how many years have you been retired?
How many years have you practiced dentistry?
What influenced your decision to serve as an adjunct faculty member? Please rank in order of importance Desire to teach dental students/Desire to give back to profession/To stay current with technology, techniques, clinical skills/CE benefits/Health insurance/Compensation/Desire to mentor dental students/To search for an associate dentist/Other
What are the barriers you see to teaching on a weekly basis? Please rank in order of importance Practice commitments/Compensation/Distance to dental school/Family commitments/Practice Debt/Student Loan Debt/Family commitments/other
What do you see as barriers for new dentists (Out of school <10 years)? Please rank in order of importance Practice commitments/Compensation/Distance to dental school/Family commitments/Practice Debt/Student Loan Debt/Family commitments/other
If the College could provide a benefit what would it be? Please rank in order of importance Student loan repayment/Health insurance/Increased compensation/Retirement benefits/Free CE courses/other
If pay was the only option that could be offered, please select an appropriate daily rate below. $100-200/$200-400/$400-600/$600+
Please select from this below your greatest perceived benefit from teaching? Passing on knowledge/Staying current/Financial compensation/Mentoring students/Interacting with colleagues/Identity with University of Iowa/Other
How many miles do you drive one way to the dental school? 0-25/26-50/51-75/76-100/101-125/126-150/151-175/176-200/200+

Descriptive analyses were completed for all variables in the sample. No statistical testing was completed due to the small nature of the study sample. All testing was performed with SAS (version 9.9; SAS Institute, Inc., Cary, NC, USA).

## Results

The total number of survey responses received were thirty three, however four surveys were incomplete thus leaving a total of twenty nine responses, giving a response rate of 63%. The sample consisted of 90% male (N = 26) and 10% female (N = 3). The age range consisted of thirty to eighty four years of age with 45% of subjects (N = 13) being less than sixty years of age and 55% of subjects (N = 16) being greater than sixty years of age. For evaluation purposes two groups were created 1) less than sixty years of age 2) greater than sixty years of age. In total, 46% of subjects were solo private practitioners, 23% in a partnership with one or more partners, and 8% were associates (non-owners) in private practice or a public health clinic. The remaining 28% comprised practitioners who were retired. A total of 24 respondents identified as general dentists with 2 identifying as endodontists (three respondents did not answer this question) (Table 2).

**Table 2:** Demographics.

Question	Percent (%)*
**Male**	90
**Female**	10
**Age**	
**25–34**	6.9%
**35–44**	10.4%
**45–54**	13.8%
**55–64**	31%
**65–74**	27.6%
**75–84**	10.6%
**Miles Driven to the School (round trip)**	
≤ 200 miles	79%
200–400 miles	21%
**How many years have you been practicing?**	
**<15**	23%
**15–30**	23%
**20+**	77%
**30+**	54%
**Hours per week you treat patients**	
**30–59 years old**	
**30–40 hours**	77%
**60+ years old**	
<20 hours	19%
**30–40 hours**	25%
**Current Practice Arrangement**	
**30–59 years old**	
Solo Practice	46.2%
Group Practice	30.8%
**60+ years old**	
Solo Practice	37.5%
Group Practice	12.5%
**Current Practice Type**	
**30–59 year old**	
General Dentistry	92.3%
Endodontics	7.7%
**60+ years old**	
General Dentistry	92.3%
Endodontics	7.7%

*percentages may not equal 100% due to rounding

Part time clinical adjunct faculty teach for a variety of reasons. When asked what influenced their decision to teach both age groups ranked teaching and mentoring dental students along with giving back to their profession and interacting with other professional colleagues as their primary reasons for teaching (Table 3). When asked about benefits that could be provided by the dental school to part time clinical adjunct faculty members, increased compensation and health insurance were the top two choices. In regards to compensation, 54% of respondents suggested $400–600 would be a fair daily rate. When separated into the two age groups, 70% of those under sixty years of age selected $400–600 when compared to only 23% of those sixty years and older selecting the same monetary range (Table 4). Barriers to teaching on a weekly basis were similar between participants in both age categories with the top three barriers being family commitments, practice commitments, and compensation (Table 3). The response was different, however, when asked the same questions but how it would apply to new dentists (those out of school less than ten years). Respondents stated the number one barrier for new dentists was student debt followed by financial compensation, practice debt, and practice commitments (Table 3). Results of survey questions can be found in Tables 2–4.

**Table 3:** Barriers and motivators to teaching.

What influenced your decision to serve as an adjunct faculty member?*	
**30–59 years old:**	**60+**
Desire to mentor dental students Desire to teach dental students Desire to give back to profession To interact with other professional colleagues To stay current with technology, techniques, clinical skills CE benefits Health insurance Compensation To search for an associate dentist	To teach dental students Desire to give back to profession To interact with other professional colleagues Desire to mentor dental students To stay current with technology, techniques, clinical skills CE benefits Compensation To search for an associate dentist Health insurance
What are the barriers you see to teaching on a weekly basis?*	
**30–59**	**60+**
Family commitments Practice commitments Compensation Committing to teaching one day a week Distance to dental school Practice debt Student loan debt Other	Family commitments Compensation Practice commitments Committing to teaching one day a week Other Distance to dental school Practice debt Student loan debt
What do you see as barriers for new dentists?*	
**30–59**	**60+**
Student loan debt Financial compensation Practice commitments Practice debt Family commitments Committing to teaching one day week Distance to school	Student loan debt Practice debt Financial compensation Practice commitments Family commitments Committing to teaching one day week Distance to school

*Ordered in most common rank given to each response by subjects

**Table 4:** Benefits.

If the college could provide a benefit what would it be?*	
**30–59**	**60+**
Health insurance Increased compensation Retirement benefits Free CE courses Student loan repayment Other	Increased compensation Health insurance Retirement benefits Free CE courses Other Student loan repayment
If pay was the only option that could be offered, please select an appropriate daily rate below*	
**30–59**	**60+**
$400–600 70% $200–400 30%	$200–400 44% $400–600 19% $600+ 13% $100–200 6% Did not respond to this question 18%

*Ordered in most common rank given to each response by subjects

## Discussion

The electronic survey tool utilized allowed part time clinical adjunct faculty members to easily fill out the anonymous survey at their convenience either at the school, their private practice, home, or on the go using a mobile device. The overall response rate of 63% is in line with other similar surveys of health care practitioners, ranging from 54–79% [9, 10]. The study design allowed subjects to privately and anonymously complete the survey to increase the validity and reliability of the questionnaire. However, validity of the survey tool is still a recognized limitation of the study. The current pilot survey will assist in validating the survey tool in ongoing collaboration with multiple international dental schools using the same survey tool. Even though the response rate was within the normal range, it may be more likely to have been completed by part time clinical adjunct faculty members who teach consistently (one half to one full day of clinical teaching in one to four quarters) instead of those who do not teach on a routine basis (occasionally teach or fill in when practitioners schedule allows) or no longer teach (retired or no longer wish to teach). Although the survey sample size is small as only one department was invited to participate in the survey at this time, it did provide information that may be applicable to all part time clinical faculty such as financial compensation, student debt, and identifying barriers to teaching and can give insight into strategies for retention and future recruitment. One limitation of the study is the lack of female respondents; however, there were 7 female faculty that met the inclusion criteria allowing for a 43% (3/7) response rate. Larger studies are needed with more female participants to increase generalizability.

In addition to the noted obstacles, travel time was an important consideration in this study. Iowa has some large and many small cities that are separated by vast rural farm lands which presents a challenge in attracting dentists to teach part time. The survey found that 52% of the current part time clinical adjunct faculty travel <100 miles round trip, 80% travel <200 miles round trip, and 20% travel 200–400 miles round trip to the school. This can be generalized to other dental schools in larger urban areas, where the mileage may not be the primary barrier, but the time it takes to travel that shorter distance in a metropolitan area. The total time it takes to travel (whether by miles or transit time) to the dental institution can cause an undue burden for the practitioner to be away from their private practice equating to lost production from the dentist and the hygiene team that may not be recuperated.

The reported decision to serve as a part time clinical adjunct faculty revolves around teaching and mentoring dental students as well as giving back to the profession. These results are in keeping with Davies et al. which found similar results [8]. It is important to note that financial compensation was not the primary driver, in fact it was consistently ranked at the bottom of the choices. When asked about barriers to teaching on a weekly basis, the number one response in both age groups was family commitments followed closely by practice commitments and compensation. In regards to asking respondents about barriers for new dentists, student loan debt was the number one reason provided by all respondents as the primary barrier for new dentists to become part time clinical adjunct faculty members. The annual ADEA survey of dental school seniors, found that graduates have an average student loan debt ranging from $129,639 to $223,984 for the classes of 2005–2015 (ranging from $0 to $550,000) [11]. Increased student debt translates into longer payment durations and balancing educational debt and living expenses. According to a survey by Nicholson et al. a total of 54% of dentists who graduated in 1996, 22.4% of 2001 graduates, and 6.6% of 2006 graduates had paid off their educational debt in 2013. It was also reported that those students who graduated with a large amount of student debt were more likely to enter private practice than to pursue graduate specialty training or to accept a government or faculty position [12]. This increased student loan debt is contributing to an increase in difficulty for dental schools to recruit new dentists (out of school less than ten years). When respondents of the current survey were asked about financial compensation, 54% of all respondents selected $400–600 as a fair daily rate; However, when broken down by age group those less than sixty years old overwhelmingly selected $400–600 (70%) while only 23% of those over sixty years old selected this option, with 44% choosing $200–400 as a daily rate in this same group. Results also show that 77% of subjects less than sixty years of age worked between 30–40 hours a week compared to only 25% of those over sixty years of age. In this latter age group, 31% worked less than 30 hours a week. Differences in compensation and hours worked are likely due to the two age groups being in different stages of dental practice. Those subjects under sixty years old likely have more practice and student debt driving their decision to work longer hours as well as motivating their choice of higher financial compensation to teach as an adjunct faculty member. With 55% of the current subjects being greater than sixty years of age and only 17% being under the age of forty five, it is also challenging for dental schools as more than half the part time faculty are nearing retirement age. This is consistent with what has been reported by the British Dental Association, with 50% of all faculty being over the age of fifty and only 10% being under the age of thirty five [13]. With 46% of dental educators being part time in North American dental schools [3], it is imperative for dental schools to recruit dentists; otherwise the part-time teaching model may need to be re-evaluated as a teaching model. The eventual loss of more senior practitioners will likely leave a knowledge gap, as supported in our study, with 54% of respondents practicing dentistry for more than 30 years and 38% practicing 35+ years. Younger faculty need to be cultivated as part time clinical adjunct faculty members early in their career, this allows them to benefit from interacting and learning from experienced full time and part time adjunct faculty in a collegial and noncompetitive environment.

## Conclusion

The current study supports the need for further research on a larger scale to help broaden the understanding of retention and recruitment of part time clinical adjunct faculty members. Taken together, the results of the current study suggest that the aging part time work force is a great concern and new part time clinical adjunct faculty members must be recruited; however, student loan debt is the primary barrier to teaching part time for new dentists. This barrier must be overcome or ameliorated in a way to make teaching less of a barrier. The primary reported reason to teach part time was to teach and mentor dental students, not for financial gain; however, part time clinical adjunct faculty members should be paid a fair wage that incentivizes them to take time away from their practice and dedicate time in their week to teach. The money earned teaching is not intended to replace lost practice income but to assist dentists in easing the financial burdens allowing them to have the choice to teach. All data used in this research is available in the supporting documents, S1 File.

## Supporting information

S1 File Copy of DescriptiveStatsByAgeGroup. (XLSX)
